# Practical Notes

**Published:** 1890-04

**Authors:** 


### PRACTICAL NOTES.

Prof. Forbes gives the class the following as a preventive against bed-sores and to harden the skin in case of fracture:

| | |
|---|---|
| Salt, - - - - - - - | ʒ iv. |
| Alum, - - - - - - - | ʒ iv |
| Water, - - - - - - - | f ℥ viij |
| Alcohol, - - - - - - | f ℥ ij M. |

Sig.—Apply over surface affected.

Dr. Van Harlingen, for a boy nine years old, with ichthyosis, directed that he be given a daily warm bath, and be anointed with goose grease or petrolatum, to keep the skin soft, and the following ointment:

| | | |
|---|---|---|
| ℞. | Sulphur. præcip., - - - - - | ʒ j. |
| | Acid. salicylic., - - - - - - | gr. xx. |
| | Adipis, - - - - - - - | ʒ j. M. |

Apply daily.

Many young children are irritable, and cry because they have intestinal flatus. Instead of using opiates, which are the basis of most of the soothing syrups, Prof. Bartholow gives the following as a valuable remedy:

| | | |
|---|---|---|
| ℞. | Misturæ assafœtidæ, - - - | f ʒ j. |
| | Sodii bromid., - - - - - | gr. iij—v. M. |

This is a dose for a child from one to four months old.—*College and Clin. Record.*

PRURITIS ANI.—

| | | |
|---|---|---|
| ℞. | Hydrarg. Chlor. Mitis, - - - | 1 drachm. |
| | Balsami Peruv., - - - - | 1 1-2 drachms. |
| | Acid. Carbolic, - - - - | 20 grains. |
| | Lanolin, - - - - - - | 1 ounce. |

**M. Sig.—Apply once or twice a day, after sponging with hot water.**

FOR PERSPIRING FEET.—

Talc, - - - - - - 10 parts.
Alum, - - - - - - 2 parts.

Largely used in Swiss army, preferable to chromic acid, and applicable even for sore feet.

Prof. Da Costa prescribed the following for a clinical patient, a girl of sixteen years, very anæmic as a result of round worms:

℞. Santonin, - - - - - - gr. ij.
Hydrarg. chlorid. mitis, - - - - gr. iv.
Sacchari lactis, - - - - - gr. iv. M.
Fiant chartæ ij.
Sig.—One evening and morning.

To destroy the ova he prescribed—

℞. Acid. carbolic., - - - - - - gr. vj.

Glycerini, - - - - - - f. ʒ j.

Aquæ, - - - - - q. s. ad. f. ʒ iij. M.

Sig.—Teaspoonful three times a day.

Also, for anæmia—

℞. Ferri et potassii tartrat., - - - ʒ iiss.

Aquæ, - - - - - - q. s. ad. f. ʒ iv. M.

Sig.—Teaspoonful three times a day.

To reduce the very high fever of typhoid fever, Prof. Da Costa advises the free use of whisky or brandy and ice to the head; sponge the patient with cold water (or, if more agreeable to patient, tepid water) three times a day. If this should not succeed, then—

℞. Antipyrin, - - - - - - gr. v.

Quinæ sulph., - - - - - - gr. j. M.

Fiat charta 1.

To be given every hour until the temperature is reduced, only two doses being required. The object of adding the quinine to the antipyrin is to prevent any depression.—*College and Clin. Record.*

In prurigo and dry eczema the following is said to be efficacious (*Medical Press and Circular* Jan. 29, 1890):

℞. Acid. carbolic., - - - - - ℥ j.-ij,
Glycerin., - - - - - q. s. to dissolve.
Syrup. aurantii, - - - - - - f. ℥ xij. M.
Sig.—Tablespoonful morning and evening.

